# Holocene ENSO variability in the South China Sea recorded by high-resolution oxygen isotope records from the shells of *Tridacna* spp.

**DOI:** 10.1038/s41598-020-61013-2

**Published:** 2020-03-03

**Authors:** Da Shao, Yanjun Mei, Zhongkang Yang, Yuhong Wang, Wenqing Yang, Yuesong Gao, Lianjiao Yang, Liguang Sun

**Affiliations:** 1 Anhui Province Key Laboratory of Polar Environment and Global Change, School of Earth and Space Sciences, University of Science and Technology of China, Hefei, 230026 China; 2 State Key Laboratory of Estuarine and Coastal Research, East China Normal University, Shanghai, 200241 China; 3 College of Resources and Environment, Key Laboratory of Agricultural Environment, Shandong Agricultural University, Tai’an, 271000 China

**Keywords:** Climate-change impacts, Palaeoclimate

## Abstract

The El Niño-Southern Oscillation (ENSO) is the principal climatic system in the modern Pacific Ocean, and it potentially influences the global climate. The South China Sea (SCS), in the western tropical Pacific, is significantly affected by ENSO activity. We have conducted a high-resolution oxygen isotope study of the shells of one modern and four fossil *Tridacna* from the Xisha Islands in the SCS. The results for the modern sample reveal that the shells of *Tridacna* are a good proxy of ENSO variability. We used the results of the oxygen isotope composition of four fossil *Tridacna* to produce high-resolution records of ENSO activity during four time slices in the Holocene. The results indicate that ENSO variability in the early Holocene was comparable to that of today, and that a minimum in the frequency and intensity of ENSO activity occurred in the mid Holocene. These findings are consistent with paleoclimatic results from corals, mollusks and sedimentary records. However, the observed extremely low frequency and moderate ENSO intensity at 4.7 ka indicate an anomalous pattern of ENSO changes within this interval of climatic transition. In addition, seasonal temperature variations during the Holocene were different from those of today and extreme seasonality may also occur during warmer periods.

## Introduction

The El Niño-Southern Oscillation (ENSO), with a typical periodicity of 2–7 years, is the most important mode of interannual changes in global climate. The impact of ENSO is global, and it influences most of the mid-low latitude climatic zones via the oceanic and atmospheric circulation system. Although instrumental records are helpful for understanding the key features of modern ENSO and its climatic impacts, its future evolution under greenhouse forcing and rapid climate change is controversial. Although the ability of several climate models to represent ENSO phenomena has been improved, several key ENSO processes remain poorly simulated^1^. In this context, paleo-ENSO records can provide complementary information to instrumental records and can help assess the performance of climate models by tracking the response of ENSO to natural climatic forcing^2^. In particular, the reconstruction of ENSO changes during the Holocene can provide valuable background information for the period prior to large-scale human activities and thus it can improve our understanding of the history and long-term evolution of ENSO.

Many ENSO reconstructions for the Holocene have been obtained from corals, mollusks, tree rings and lake sediments^3–11^. However, reported results of ENSO variability during the Holocene are controversial. ENSO reconstructions based on lacustrine sediments from southern Ecuador indicated a general increase of ENSO variance from the early to late Holocene and weak ENSO activity during the early Holocene^9,12^. Cobb *et al*.^6^ synthesized multiple ENSO reconstructions based on δ^18^O records from fossil corals and highlighted highly variable ENSO activity during the Holocene, which contradicts the results of previous studies. A later mollusk-based study of Peru^3^ also reported variable ENSO changes during the early Holocene, comparable with the late Holocene, and contradicted the hypothesis of limited ENSO variability before 5 ka. A recent study reanalyzed the lacustrine sedimentary record of Laguna Pallcacocha in Ecuador and suggested that the Holocene flooding record of the site was not a reliable record of past El Niño behavior^13^, which is also indicated by several other ENSO reconstructions^5,6,11,14^. In addition, various studies revealed a mid-Holocene minimum of ENSO variance in the tropical Pacific region^5–8^ though we cannot exclude the possibility of spatial variations^15^. Clearly, therefore, more high-resolution records are needed to determine the history of ENSO activity in the Holocene.

The South China Sea (SCS) lies on the edge of Western Pacific Warm Pool (WPWP) which is substantially affected by ENSO^16^. Generally, temperatures are warmer and precipitation is weaker during El Niño conditions, and vice versa during La Niña conditions. Other climate systems, such as the Intertropical Convergence Zone (ITCZ)^17^, the Indian Ocean Dipole Mode Index (DMI)^18^ and East Asian monsoon^19^, may also impact this region. Biogenic carbonates like coral or the bivalve *Tridacna* spp. are ideal marine archives for reconstructing high-resolution climate records^20–22^.

*Tridacna* spp. occur mainly around the tropical Pacific and Indian Oceans. It is climatically sensitive and thus is well suited for paleoclimatic research^23,24^. Previous studies have shown that the δ^18^O values of the aragonite of *Tridacna* provide a record of both SST and SSS^23,25–27^, while element ratios such as Sr/Ca and Mg/Ca could be used as proxies of changes in SST^23,28–30^. Stable isotope or element data at a bimonthly or monthly resolution can be determined from the shells of *Tridacna*^28,31^; moreover, with high growth rates or the application of optimal technical methods, a daily resolution can be achieved^27,30,32^. Analyses of *Tridacna* have been successfully used to reconstruct ENSO variations for different time periods, such as the present^33^, the early Holocene^31^, Pleistocene^28^ and Miocene^34^. In addition, the excellent preservation of the shell of *Tridacna* enhances its usefulness for paleoclimatic research.

**Figure 1:**
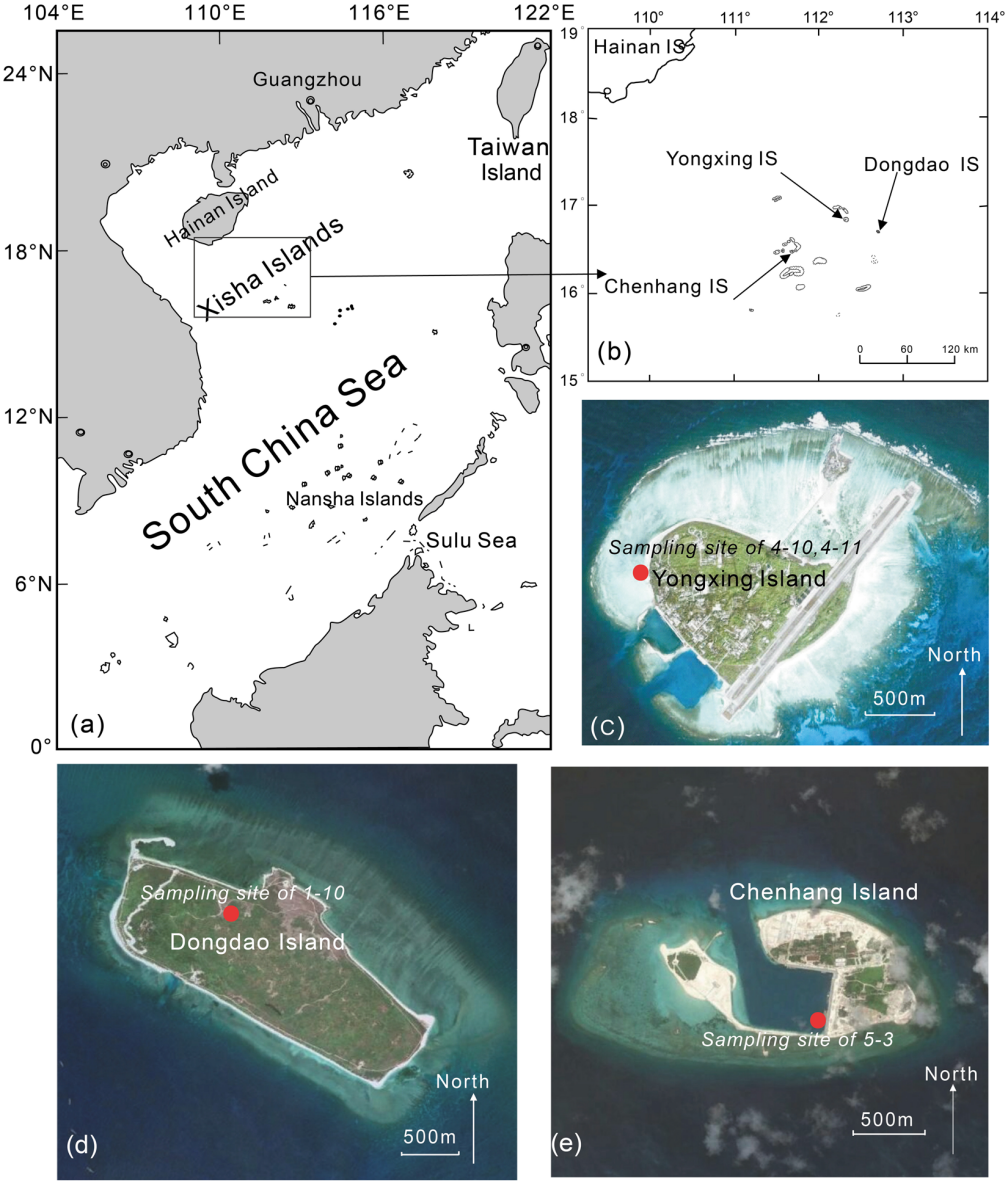
Map of the sampling sites. (**a**) South China Sea; (**b**) Xisha Islands; (**c**) Yongxing Island (sites 4–10 and 4–11); (**d**) Dongdao Island (site 1–10); (**e**) Chenhang Island (site 5–3). The high-res images shown in (**c–e**) are exported from Google Earth Pro on desktop and DigitalGlobe is the data provider.

During field work in 2012 and 2013, we collected one modern and four ancient shells of *Tridacna* from the Xisha Islands in the SCS (Figs. 1 and S1). In the present study, we analyzed several climatic proxies of the five specimens of *Tridacna* with the aim of evaluating variations in ENSO and climatic seasonality in the SCS during the Holocene.

## Results

The results of AMS ^14^C dating for the 5 samples of *Tridacna* are given in Table 1. The calibrated ages of the four ancient *Tridacna* span the interval of 7.6 ka-2.7 ka.

**Table 1:** Dating results of five samples of *Tridacna*.

UGAMS#	Sample ID	Sampling site	Material	Conventional ^14^C age (years BP)	Cal. Year BP (two sigma range)
18964	XSN	By fishermen	carbonate	modern	modern
12186	1–10	Dongdao Island	carbonate	2550 ± 25	2697–2749
13177	4–11	Yongxing Island	carbonate	4160 ± 25	4610–4768
13176	4–10	Yongxing Island	carbonate	6050 ± 30	6825–6981
13185	5–3	Chenhang Island	carbonate	6720 ± 25	7562–7623

UGAMS# is the laboratory number of the University of Georgia and the sample ID is the number of the sample. All dated materials are carbonates.

**Figure 2:**
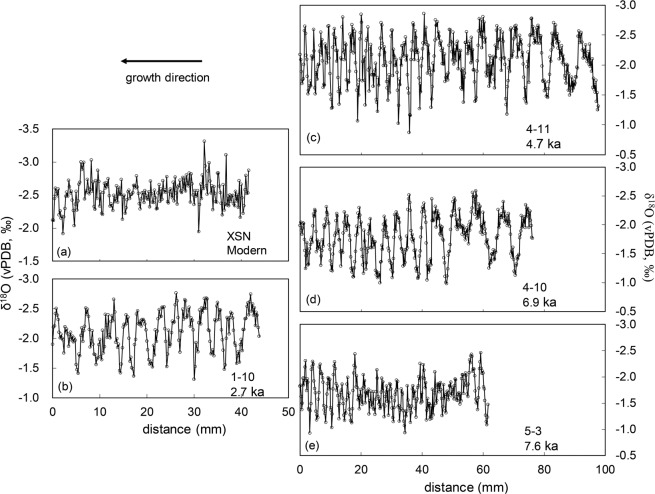
δ^18^O records of the five *Tridacna* samples. (**a**) XSN; (**b**) 1–10; (**c**) 4–11; (**d**) 4–10; (**e**) 5-3. The growth direction is from right to left, and the distances are from the interior of the shell.

For the modern *Tridacna* sample, the δ^18^O values of 209 data points range from −3.31‰ to −1.92‰, with an average of −2.25 ± 0.20‰. These δ^18^O values are more negative than those from other *Tridacna* sample used in our previous study^23,35^, and this is caused by several outliers of δ^18^O values for the modern *Tridacana* XSN (Fig. 2a). The results are plotted along the growth direction in Fig. 2a, and the record exhibits an obvious yearly cyclicity (SI material 1). Since the *Tridacna* specimen was collected in early 2013, the age of the end of the record is January or February of 2013, which provides the basis for the subsequent chronology.

The δ^18^O profiles of the four fossil *Tridacna* samples are shown in Fig. 2b–e. The δ^18^O values of sample 1–10 (220 data points) range from −2.76‰ to −1.31‰ with a mean of −2.02 ± 0.33‰ (Fig. 2b); those of sample 4–11 (490 data points) range from −2.86‰ to −0.87‰ with a mean of −2.08 ± 0.38‰ (Fig. 2c); those of sample 4–10 (379 data points) range from −2.59‰ to −0.98‰ with a mean of −1.79 ± 0.36‰ (Fig. 2d); and those of sample 5–3 (309 data points) range from −2.46‰ to −0.93‰ with a mean of −1.68 ± 0.31‰ (Fig. 2e). Like the modern sample, all four fossil samples exhibit clear annual cycles. It is noteworthy that the standard error of δ^18^O may be influenced by random sampling, V_T_ (the variation of annual mean temperature) is overestimated when using short records^36^, and much longer records are required to estimate ENSO variance^37^. According to Carré *et al*.^36^, in order to make the standard error of V_T_ less than 1, the year length should be more than 10 years, and the error can be reduced to ~0.84 for the 15-year-long records. Therefore, sample 4–11 (4.7 ka, 25 yrs) and 5–3 (7.6 ka, 30 yrs) are long enough to estimate ENSO variations. Sample 1–10 (2.7 ka, 12 yrs) and sample 4–10 (6.9 ka, 14 yrs) may be not long enough for robust ENSO estimates, but their standard errors are still under 1.0 (0.94 and 0.86, respectively). Thus more individual records will provide more robust results and reduce uncertainties.

Given the long summer and short winter in the study region, we assumed that the maximum value of each cycle corresponded to January. Based on this, we identified the annual cycles and discarded data from both ends of the record, which did not exhibit a complete annual cycle. The annual cycles for the modern and 5–3 sample are not as clear as samples 1–10, 4–11 and 4–10. Thus the age models for the modern and 5-3 sample were mainly reconstructed based on yearly cyclicity visible by eyesight and comparison between the δ^18^O profile and the *Tridacna* annual banding (SI material 2). Interpolation was then applied to the oxygen isotope data between two adjacent maxima to produce 12 data points, representing a monthly resolution, for each year. In order to avoid the effect of growth rate, the oxygen isotope data was detrended using linear regression. Finally, we normalized the revised oxygen isotope data as Z values^38^. The results are shown in Fig. S2.

## Discussion

### δ^18^O of modern Tridacna as a proxy for ENSO

The anomalies in oxygen isotope data are typically used as an index of ENSO variability^28,31^. The δ^18^O of *Tridacna* is influenced by both SST and the salinity of seawater; however, for *Tridacna* in the Xisha Islands, SST is regarded as a much more important influence than seawater δ^18^O, which has been quantified based on the modern sample from this locality^23^. Our modern *Tridacna* sample was collected in early 2013, and its record has a monthly resolution. We compared the instrumental SST data for the Xisha Islands (obtained from NOAA) with our oxygen isotope dataset (Fig. S3) and performed correlation analysis. The resulting correlation coefficient was −0.44 (n = 205, p < 0.0001) and thus the correlation is significant. In addition, another modern *Tridacna* in the SCS yielded a much stronger correlation (r = −0.82) between δ^18^O and SST^35^. The correlation between the δ^18^O of *Tridacna* and local SST reveals the important control of SST on the inter-annual variation of the δ^18^O records^35^.

**Figure 3:**
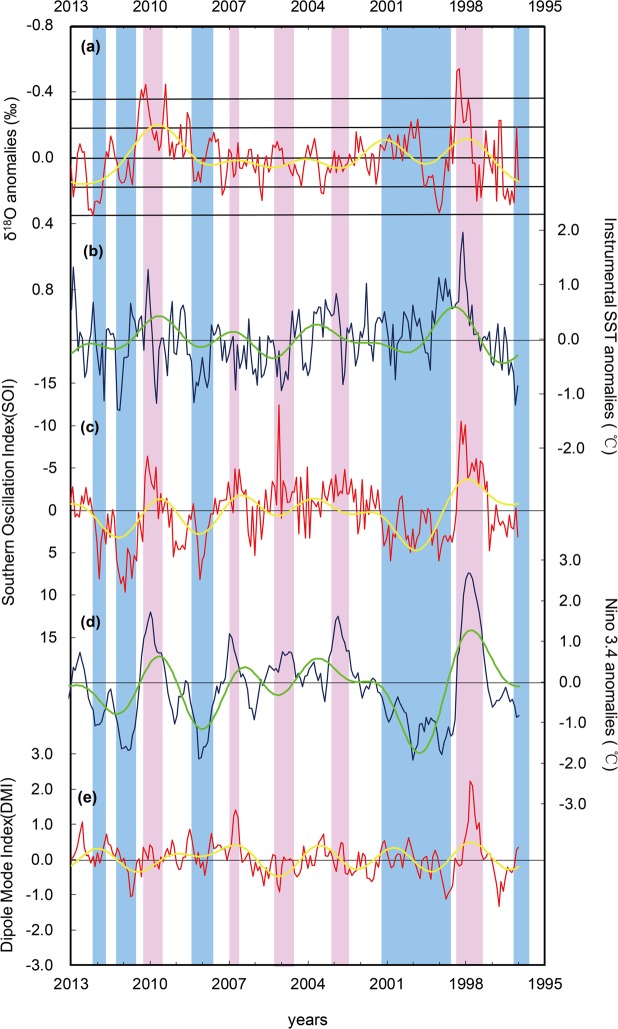
Comparison and analysis of modern *Tridacna* (XSN) δ^18^O anomalies, instrumental SST, SOI, anomalies of Nino 3.4 and DMI. (**a**) δ^18^O anomalies for XSN (red line) and results of band-pass filtering (2.5–7 years, yellow line). The anomalies were calculated by using the monthly resolution data minus the mean monthly value for that period. The dashed line is the threshold value for defining an ENSO event and the point-dashed line is the threshold for defining a strong ENSO event. (**b**) Instrumental SST anomalies (blue line) and 2.5–7-year band-pass filter results (green line). SST anomalies are the monthly SST minus the corresponding mean monthly SST; SST date are from NOAA. (**c**) SOI (red line) and 2.5–7-year band-pass filter results (yellow line); the SOI data are from http://www.cgd.ucar.edu/cas/catalog/climind/soiAnnual.html. (**d**) Nino 3.4 SST anomalies (blue line) and 2.5–7-year band-pass filter results (green line); the data are from Nino3.4 ANOM http://www.cpc.ncep.noaa.gov/data/indices/sstoi.indices. (**e**) Indian Ocean Dipole Mode Index (DMI, red line) and 2.5-7-year band-pass filter results (yellow line). DMI date was obtained from the monthly DMI http://www.jamstec.go.jp/frsgc/research/d1/iod/iod/dipole_mode_index.html. The red shading represents El Niño events and the blue shading represents La Niña events, based on the Oceanic Niño Index (ONI) http://www.cpc.ncep.noaa.gov/products/analysis_monitoring/ensostuff/ensoyears.shtml.

We calculated the anomalies of the oxygen isotope records by subtracting the monthly mean from the adjusted isotope data (Fig. 3a). Band-pass filtering was performed to extract periodic components with wavelength of 2.5–7 years, which potentially represented ENSO signals. The instrumental SST data for the Xisha Islands (Fig. 3b), Southern Oscillation Index (SOI, Fig. 3c), SST in Nino 3.4 (Fig. 3d), and Dipole Mode Index (DMI, Fig. 3e) were processed in a similar way. The δ^18^O anomalies show similar variations to the anomalies of the instrumental SST data (r = −0.17, p < 0.05) for the Xisha Islands, SOI (r = 0.17, p < 0.05) and SST in Nino 3.4, whereas they are clearly different to the anomalies of the DMI (Fig. 3). Therefore, the interannual climate variability of the South China Sea is mainly controlled by ENSO^18,21^.

We regard the oxygen isotope anomalies as a good proxy for ENSO variations. A negative value represents an El Niño event (EN), and a positive value a La Niña event (LN). Compared with the instrumental SST anomalies, SOI and Niño 3.4 SST anomalies, the oxygen isotope anomalies exhibit a clear and strong signal for the EN of 1998 and 2010 and the LN of 1999 and 2012. The signals for LN of 2008 and 2011 are also very clear, although their amplitude is comparatively low. For LN of 1996, the signals exhibit a slight offset, but for EN of 2003, 2005, and 2007, the signals are unclear. We conclude that, overall, the oxygen isotope anomalies reflect ENSO events and to some degree also the ENSO magnitude.

NOAA has proposed the use of a primary indicator, the Oceanic Nino Index(ONI), for monitoring El Niño and La Niña. ONI is defined as the ~3 month moving average of Niño 3.4 SST anomalies. An ONI above +0.5 is regarded as indicative of El Niño conditions, and an ONI below −0.5 of La Niña conditions. According to ONI, 10 ENSO events occurred during this interval. To define ENSO events from the oxygen isotope datasets, we defined a similar index, called the oxygen isotope anomaly index (OIAI). As the δ^18^O anomaly of our modern one is 0.00 ± 0.16‰, we chose 0.16, the standard deviation of δ^18^O anomalies of the modern *Tridacna* sample, as the threshold value. The threshold value for the modern *Tridacna* YX1 (also collected in the Xisha Islands) used in our previous study^23,35^ is also 0.16; however, the threshold value is 0.15 for giant clams (Tridacna sp.) from Papua New Guinea^31^. Thus, the threshold value is very likely related to the sampling locations and using 0.16 as the threshold value for samples near Xisha Islands is appropriate. In this study, an OIAI below −0.16 for six consecutive months is regarded as an unambiguous indicator of an ENSO event, and a value below −0.32 of a strong ENSO event. According to this definition, EN of 1997–1998 and 2009–2010 and LN of 1995–1996 and 2011–2012 can be identified by OIAI (Fig. 3). For LN of 1998–2001, OIAI does not exceed 0.16 for six consecutive months, but it does exceed 0.1 for ten consecutive months and has a maximum value above 0.32 (Fig. 3); thus, we regard it as indicating an ENSO event. The application of OIAI to our datasets enables us to identify five relatively ENSO events during the past 17 years, with a corresponding frequency of 5/17. However, the ENSO events of (2002–2003, 2004–2005, 2006–2007, 2007–2008, 2010–2011) determined by ONI were not recorded, which could be related to weak signals of these events. Considering that the strong ENSO events determined by OIAI are clearly recorded in the local instrumental SST records, while the weak ENSO events cannot be observed in the local instrumental SST records. Therefore, as the study area is located at the western margin of the Pacific, weak ENSO events may not have a strong influence in the study area and we regard OIAI as a reliable proxy for identifying relatively strong ENSO events in the Xisha Islands.

### ENSO changes during the Holocene

**Figure 4:**
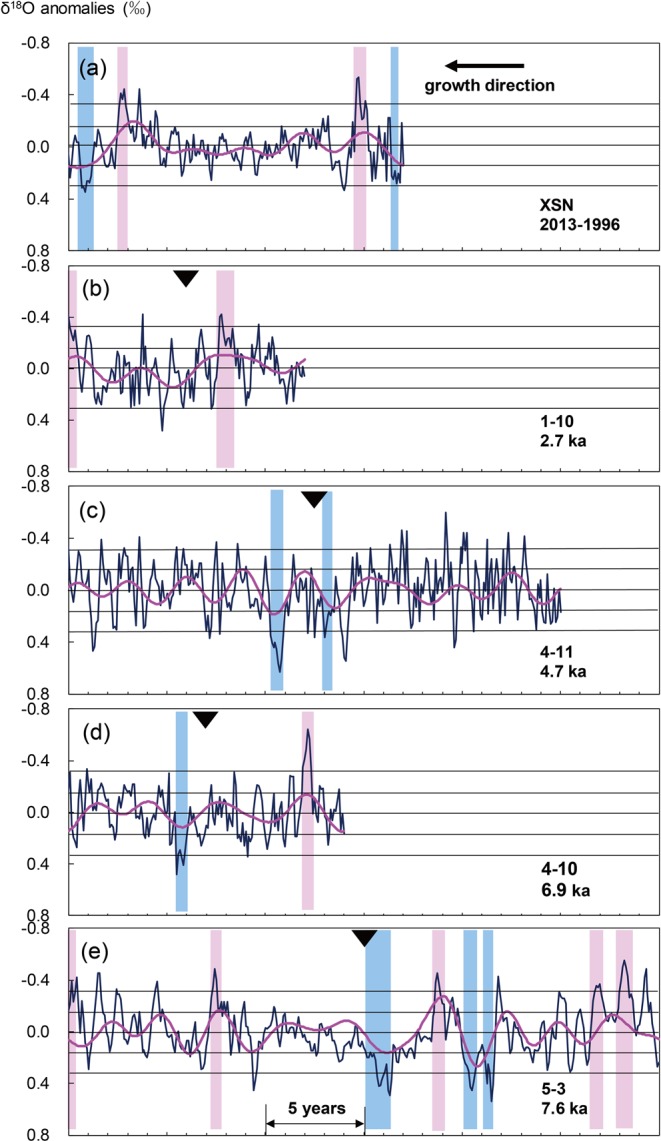
ENSO signals shown as δ^18^O anomalies for the modern *Tridacna* sample and for four Holocene *Tridacna* samples. From the top: XSN (**a**), 1–10 (**b**), 4–11 (**c**), 4–10 (**d**), 5-3. (**e**) The blue lines represent monthly δ^18^O anomalies and the red lines are 2.5-7-year band-pass filter results. The dashed lines and point-dashed lines represent the ENSO event thresholds of ±0.16 and ±0.32. The red shading indicates El Niño events and the blue shading indicates La Niña events which exceed the threshold of 0.16 for six consecutive months. Black triangles are the approximate positions of samples used for dating.

According to the above discussion, an OIAI below −0.16 for six consecutive months is used to define an unambiguous ENSO event, and a value below −0.32 define a strong ENSO event. We examined the ENSO frequency and intensity of four Holocene *Tridacna* samples (Fig. 4), as inferred from OIAI, using the method described in section 4.1. Sample 1–10 (2.7 ka, 12 yrs) has 2 EN with an occurrence frequency of 2/12 (16.7%); sample 4–11 (4.7 ka, 25 yrs) has 2 LN with a frequency of 2/25 (8%); and sample 4–10 (6.9 ka, 14 yrs) has 1 EN and 1 LN with a frequency of 2/14 (14.3%). Finally, specimen 5–3 (7.6 ka, 30 yrs) has 5 EN and 3 LN; however, the interval between the last 2 LN and 2 EN is short and there is no reversal of positive or negative anomalies. For this reason, we merged these two events, which results in 4 EN and 2 LN with a frequency of 6/30 (20%). Compared to the ENSO frequency of the modern *Tridacna* sample (5/17≈29.4%), the frequency is relatively low for the four fossil *Tridacna* specimens. Overall, the frequency of ENSO events in our *Tridacna* samples decreases from 7.6 ka onwards, reaches the lowest levels around 4.7 ka, and then increases to the highest levels in modern times.

We also evaluated the amplitude of ENSO changes based on the standard deviation of the 2.5–7 yr band-pass-filtered time series of the 1 modern and 4 fossil *Tridacna* samples (Fig. S4), which eliminates periodicities which are either too long or too short. The total variance of the 2.5–7 yr band-pass filtered results provides a measure of the amplitude of ENSO variations and hence its intensity^10^. Compared with the modern *Tridacna* sample, samples 1–10 and 4–11 show no obvious difference, whereas the variance of sample 4–10 is 31% lower (F-test, 99%), and that of sample 5–3 is 28% higher (F-test, 99%). Therefore, the intensity of ENSO changes was moderate at ~4.7 ka (Fig. S4), although the frequency of ENSO events was extremely low during this period. Since 4.7 ka was a transitional period from the mid- to the late Holocene, the climatic instability at this time may have led to abnormal fluctuations in ENSO. Overall, the variance trend of our samples during the Holocene is similar to the paleo-ENSO records based on fossil corals^6^, which show a significant reduction in ENSO variance in the mid-Holocene.

In summary, a mid-Holocene minimum in the frequency and intensity of ENSO activity is observed in our study, which supports the results from coral, mollusk and sedimentary records from the tropical Pacific region which also show a reduction in ENSO variance at this time^3,5,7,11^. A synthesis of coral δ^18^O records from the equatorial Pacific indicates a reduced mid-Holocene ENSO variance^6^, similar to that observed in the δ^18^O record of fossil mollusk shells from Peru^3^. In addition, estimates of ENSO variability from stalagmite δ^18^O variance (BA03) in the western equatorial Pacific indicate reduced ENSO activity during 3.5–6.5 ka^5^; and the δ^18^O record of planktonic foraminifera (*G. ruber*) in sediment core V21–30 from the Galápagos region also records an interval of minimum ENSO variance during 4–6 ka^14^. Many recent studies document a mid-Holocene minimum in ENSO variance and a potential driving mechanism has been proposed. Chen *et al*.^5^ suggested that persistent convective activity during the mid-Holocene led to enhanced Walker circulation, intensified easterly winds, and upwelling in the eastern equatorial Pacific, which thereby suppressed the development of El Niño events and thus ENSO variance. In addition, McGregor *et al*.^8^ suggested that zonal winds and SST gradients in the tropical Pacific were enhanced during the mid-Holocene in response to higher boreal summer insolation, which could have suppressed ENSO activity. These proposed mechanisms are consistent with subsequent modelling studies^39^.

In addition to the mid-Holocene minimum of ENSO variance, our data provide more information about ENSO activity in the western equatorial Pacific during the early Holocene. Our results indicate a moderate level of ENSO variance in the early Holocene, close to the modern level, which is in good agreement with ENSO variance reconstructed from fossil mollusk shells from Peru^3^. Although a sedimentary record from Laguna Pallcacocha in southern Ecuador indicated a very low level of ENSO activity during the early Holocene^9^, a recent study which reanalyzed the sediments from multiple cores from the site concluded that the sedimentary record is not a reliable record of ENSO activity^13^. In addition, paleoclimatic records from corals, mollusks and foraminifera obtained in recent years all indicate that ENSO was more active in the early Holocene than in the mid Holocene^3,5–7,14^.

Coral records from the Northern Line Islands^6^ demonstrated that although ENSO variance during the 20^th^ century was higher than the average level during the Holocene, it was not unprecedented, which is consistent with our results. It is noteworthy that the amplitude of ENSO variance in our modern *Tridacna* sample is not significantly higher than that in the early and late Holocene, as revealed by our fossil *Tridacna* specimens; nevertheless, the frequency of ENSO events in the modern *Tridacna* sample is significantly higher than that in the fossil *Tridacna* samples. This may be related to the less clearly defined periodicities, hence seasonality, in the modern *Tridacna* sample.

According to the profile of δ^18^O anomalies in the 7.6 ka *Tridacna* sample (Fig. 4e), two of the typical LN events exceed the threshold for defining a strong ENSO event, and they may represent extreme cold events. This phenomenon is not unique: in the modern *Tridacna* sample, two strong LN events are evident in the modern warm period, which represent abnormal global climate changes. In the 4.7 ka *Tridacna* sample (Fig. 4c), a LN event is evident which exceeds the threshold for defining a strong ENSO event; however, there is no evidence of such an event during a cold period such as at 2.7 ka. The interval at ~7.6 ka was a representative warm period during the Holocene Thermal Maximum, while 4.7 ka was a transitional period between the mid- to late Holocene. Therefore, extreme cooling events and climatic fluctuations may have occurred not only during periods of climatic transition, when the climate was unstable, but also during warm periods.

### Seasonality of the δ^18^O records of modern and Holocene Tridacna

Although the oxygen isotope composition of *Tridacna* is influenced by both SST and seawater δ^18^O, the former has been shown to be the main influencing factor^23^; thus the oxygen isotope record of *Tridacna* can be used as proxy of climatic seasonality. Notably, all five *Tridacna* records exhibit clear annual cycles (Fig. S2).

**Figure 5:**
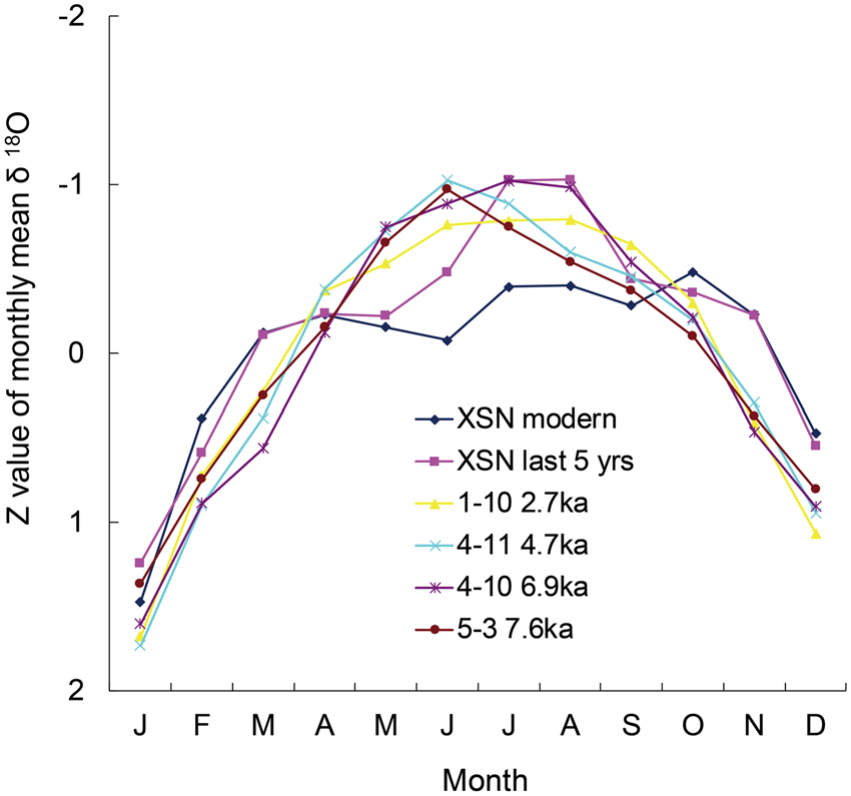
Normalized monthly mean oxygen isotope values (Z value) for each *Tridacna* sample. The last five years of the modern *Tridacna* exhibit a better defined periodicity and are included in the figure.

The monthly mean δ^18^O value was used to reflect average seasonal changes within a specific time interval (Fig. 5). As the periodicity evident in modern *Tridacna* before maturity is relatively poorly defined, we selected the monthly mean value of the last five years to represent the seasonality of modern *Tridacna*. To compare the seasonality between different time periods, Z values were calculated by subtracting the mean value for a specific time period from the monthly mean δ^18^O, and then dividing the result by the standard deviation.

Given the long summer and short winter in the study region (Fig. S3), we assumed that the maximum oxygen isotope value corresponded to January. Based on the above assumption, the winter and summer seasons could be easily distinguished. For modern *Tridacna*, June, July and August correspond to the summer season, which is consistent with the instrumental records. For each fossil *Tridacna* sample, there is a little difference for the summer season at different periods, though the difference may not exceed one month. Therefore, when considering SST variations, the difference of seasonality during the Holocene is not evident in the study.

The difference between the maximum and minimum values of the normalized monthly data (Fig. S2) may reflect extremes in seasonality. The modern *Tridacna* sample has maximum and minimum values of 3.186 and −3.260, respectively, with a range of 6.446. For the fossil *Tridacna* samples, the ranges are 4.560 (sample 1–10), 5.018 (sample 4–11), 4.119 (sample 4–10) and 5.276 (sample 5–3). Thus, the range is greater in the modern *Tridacna* sample and that of 7.6 ka, compared to the other samples, which indicates a greater amplitude of climatic variability at these times.

From the foregoing, we conclude that the seasonal changes varied significantly during the Holocene. Deng *et al*.^20^ observed a similar phenomenon and reported phase differences between the rainy season and the SST record. Here, we consider SST as the main driver of changes in δ^18^O. The modern period and 7.6 ka are typical warm periods. Compared with the seasonal changes at 6.9 ka (sample 4–10), those of today (sample XSN) and at 7.6 ka (sample 5-3) were characterized by higher winter SST and equivalent summer SST (Fig. 5). 2.7 ka was a relatively cool interval and sample 1–10 indicates relatively cool SSTs during both summer and winter (Fig. 5). In addition, the extreme seasonal changes observed in modern times and at 7.6 ka are of higher amplitude than during the other studied intervals, which indicates that extreme seasonality may also occur during these warm periods.

## Conclusions

We have obtained high-resolution oxygen isotope records from five specimens of *Tridacna* from the Xisha Islands, in the SCS, which span intervals from modern to the Holocene. Changes in δ^18^O anomalies in the records are closely linked to ENSO variations, with a negative anomaly corresponding to an El Niño event and a positive anomaly to a La Niña event. The δ^18^O record of the modern *Tridacna* specimen captures the main El Niño and La Niña events during 1996–2013 and is consistent with local instrumental records. The results for the four fossil *Tridacna* specimens reveal that ENSO variance in the early Holocene was comparable to that of today, and that ENSO activity was significantly reduced during the mid-Holocene, in line with coral and mollusk records. However, the extremely low frequency and moderate amplitude of ENSO changes at 4.7 ka indicate the occurrence of anomalous ENSO changes during this climatic transition. Moreover, there were differences for the summer season time during the Holocene in the SCS and extreme seasonality may also occur during warmer periods. Overall, our results provide important information about ENSO variability in the SCS during the Holocene.

## Materials and Methods

### Materials

The Xisha Islands are located in the northwestern part of the South China Sea (15 °40′–17 °10′N, 111°–113 °E, Fig. 1a). They have an annual mean temperature of 26–27 °C and annual mean precipitation of 1500 mm, and a clear separation of wet and dry seasons. The rainy season is from June to November, when the southwest monsoon prevails, with maximum temperatures of up to ~30 °C and precipitation of 1100 mm; during the dry season, from December to May, the northeast monsoon prevails, and the minimum temperature is 24 °C and the precipitation is ~400 mm. The Xisha Islands lie on the edge of the WPWP, a typical tropical marine area affected by ENSO. The Xisha Islands mainly consist of carbonate islets constructed by coral and are covered with well-developed tropical vegetation^40,41^.

*Tridacna* is a specialized genus of bivalvia and has a symbiotic relationship with zooxanthellae. All five samples of *Tridacna* spp. are more than 400-mm thick and have dense annual layers (Fig. S1). The samples were collected in 2012 from the beach or from the cementitious coral reefs of Yongxing Island (samples 4–10, 4–11), Dongdao Island (sample 1–10) and Chenhang Island (sample 5-3) (Fig. 1c–e). A specimen of living *Tridacna* (XSN) was obtained by fishermen from the Xisha Islands in 2013. The ages of these fossil Tridacnidae span from 7600 BP to 2700 BP and these giant fossil Tridacnidae on reef flats were likely transported to Xisha Islands due to the AD 1076 tsunami in the South China Sea^42^.

## Methods

Each specimen was cut in half along the maximum growth axis, and a 1-cm-thick slice was cut out and carefully washed with deionized water (Fig. S1). From each slice, a vertical strip was cut out along the growth line and ground into a powder for X-ray Powder Diffraction (XRD) analysis. The results showed that the specimens of *Tridacna* were mainly composed of aragonite and were not influenced by diagenetic recrystallization.

### AMS^14^C dating

As fossil *Tridacna* cannot provide independent geochronological control using either closed or open-system U-series dating techniques^43^. Therefore, we used Accelerator Mass Spectrometer radiocarbon (AMS^14^C) dating to determine the ages of the *Tridacna* samples. More than 100 mg of powder samples were taken from the middle section of the slices, and dating was performed at the Center for Applied Isotope Studies at the University of Georgia, U.S.A. The modern *tridacna* spp. (XSN) was dated as modern, indicating lack of reservoir effects. In addition, Yan and Liu^44^ preformed radiocarbon dating analysis for 20 modern *tridacna* from Xisha Islands, South China Sea, and also found no reservoir effects, the AMS ^14^C ages were calibrated using Calib 7.0.2^45^ without further correction for reservoir effects.

### ^18^O measurements

After ultrasound washing of the cut strip, we collected subsamples of ~1 mg using a microdrill at an interval of 0.2 mm. Strips that were too long to be placed on the microdrill platform were separated into two sections before obtaining subsamples. The experiments were performed using a Thermo-Finnigan MAT 253 with Kiel IV Carbonate Device at the South China Sea Institute of Oceanology, Chinese Academy of Sciences; and with a Thermo-Finnigan MAT 252 with Kiel III Carbonate Device at the Institute of Earth Environment, Chinese Academy of Sciences. About 100 µg of subsample was used for the oxygen isotope analysis using the MAT 253 (samples XSN, 4–10, 5-3) and MAT 252 (samples 1–10, 4–11) which were calibrated using vPDB and monitored by the control samples NBS18. The standard deviations for δ^18^O analyses are ±0.05‰ (MAT253) and ±0.1‰ (MAT252).

## Supplementary information

Supplementary material.
