# Supplementary material for: Holocene ENSO variability in the South China Sea recorded by high-resolution oxygen isotope records from the shells of Tridacna spp
Source: Sci Rep. 2020 Mar 3;10:3921. doi: 10.1038/s41598-020-61013-2 (PMC7054325; doi:10.1038/s41598-020-61013-2)

Supporting Information for

Holocene ENSO variability in the South China Sea recorded by high-resolution oxygen isotope records from the shells of *Tridacna* spp.

**Da Shao^1^*^,†^, Yanjun Mei^1,2,†^, Zhongkang Yang^1,3^*^,†^, Yuhong Wang^1^, Wenqing Yang^1^, Yuesong Gao^1^, Lianjiao Yang^1^ and Liguang Sun^1^***

^1^ Anhui Province Key Laboratory of Polar Environment and Global Change, School of Earth and Space Sciences, University of Science and Technology of China, Hefei, 230026, China

^2^ State Key Laboratory of Estuarine and Coastal Research, East China Normal University, Shanghai, 200241, China

^3^ College of Resources and Environment, Key Laboratory of Agricultural Environment, Shandong Agricultural University, Tai'an, 271000, China

* Corresponding author: Liguang Sun ([slg@ustc.edu.cn](mailto:slg@ustc.edu.cn)), Da Shao ([shaoda@ustc.edu.cn](mailto:shaoda@ustc.edu.cn)) and Zhongkang Yang ([zkyang@mail.ustc.edu.cn](mailto:zkyang@mail.ustc.edu.cn))

† These authors contributed equally to this work.

**Figure S1**


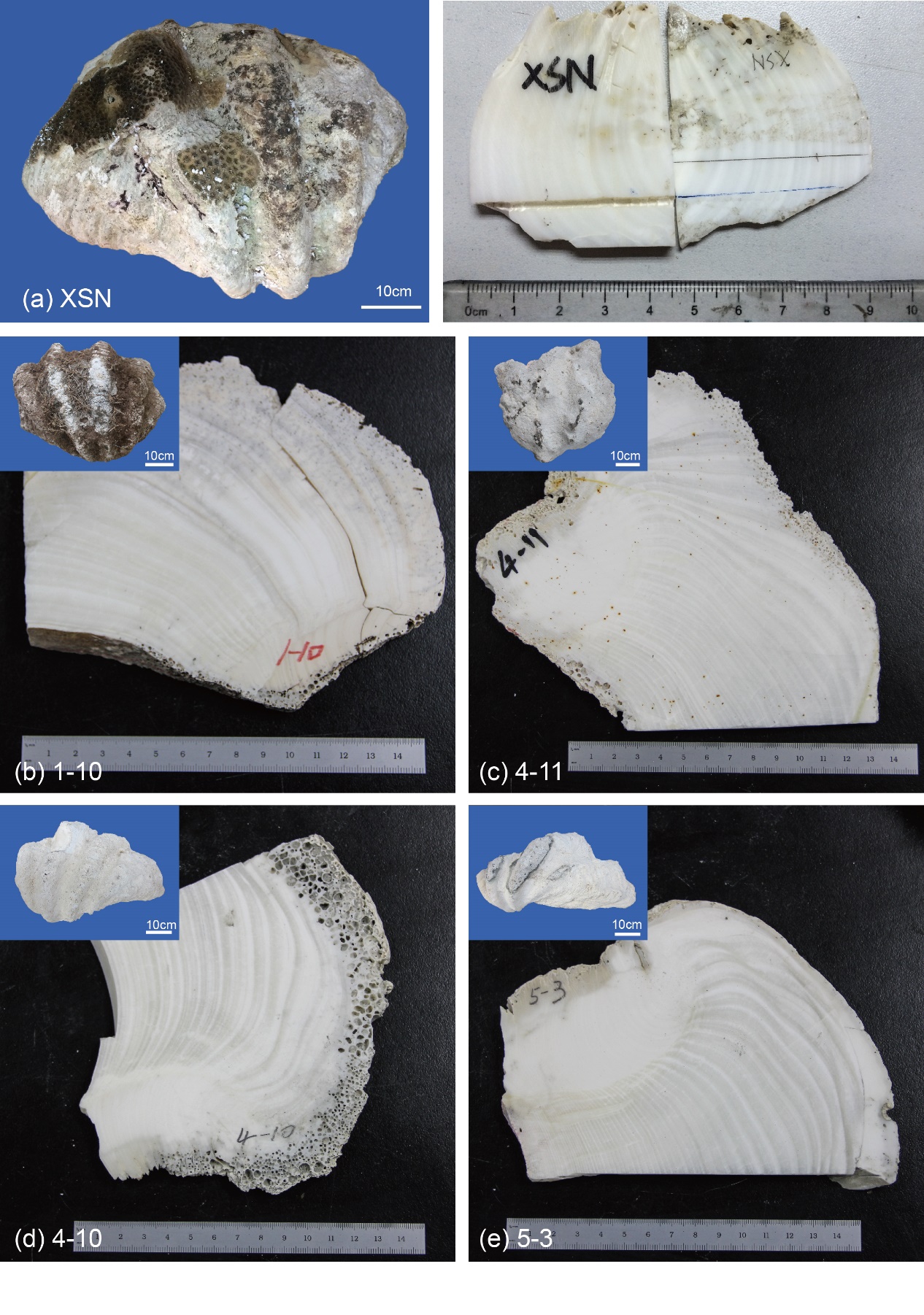


**Figure S1.** Photos of the five *Tridacna* samples from the Xisha Islands. SCS. (a) XSN; (b) 1-10; (c) 4-11; (d) 4-10; (e) 5-3. The larger photos show the section of each *Tridacna* sample and the smaller photos in the top left corner show the appearance of *Tridacna* with a scale of 10 cm. The right photo of XSN (a) shows the same sections.

**Figure S2**


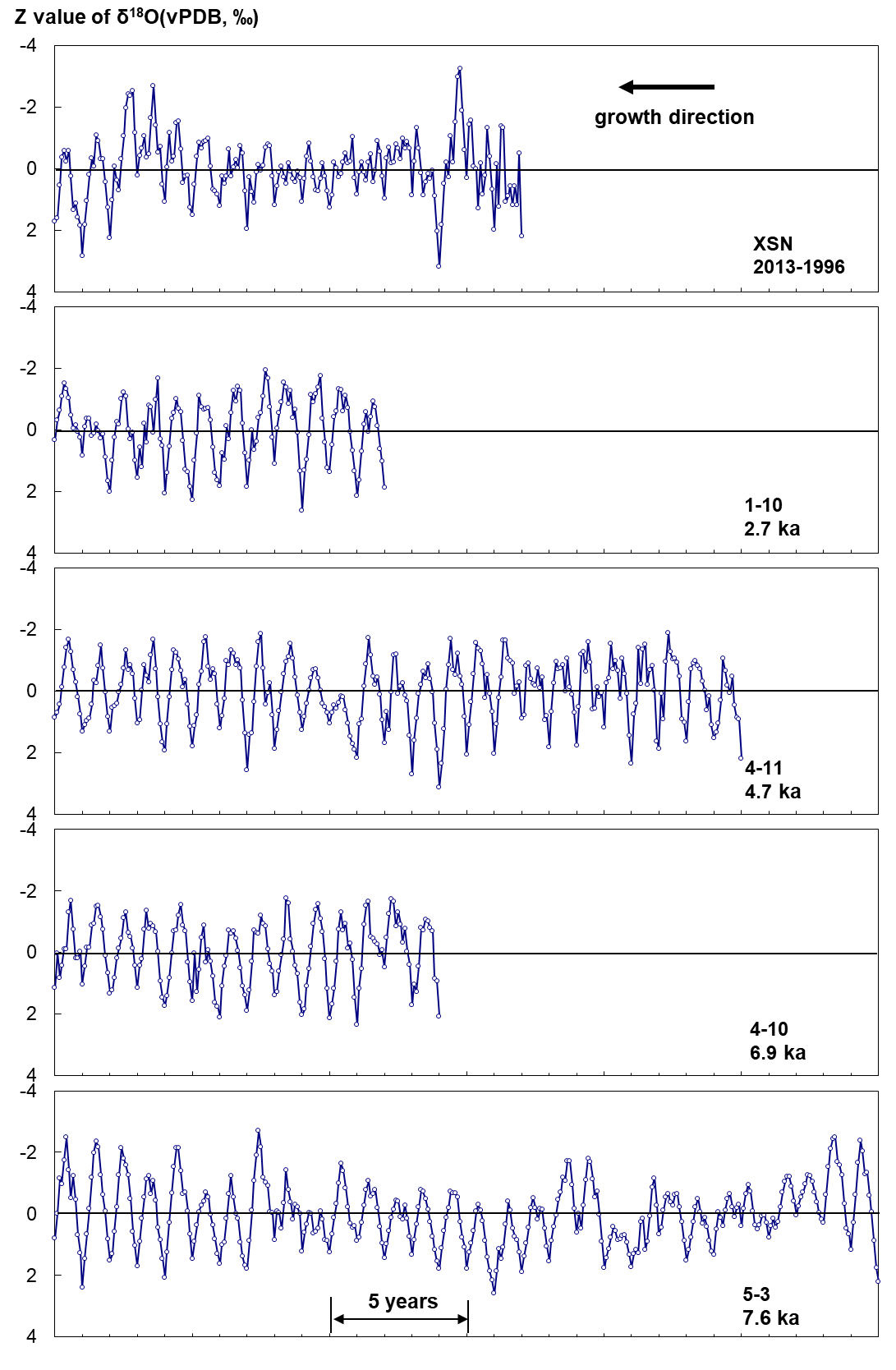


**Figure S2.** Normalized δ^18^O records of *Tridacna* (Z values). (a) XSN; (b) 1-10; (c) 4-11; (d) 4-10; (e) 5-3. The growth directions are from right to left. The minor ticks on the X axis represent 1 year.

**Figure S3**

**Figure S3.**

Temporal variations in monthly mean δ^18^O values of XSN (blue) and local instrumental SST (pink). The records are significantly correlated (n=205, r=-0.44, p<0.0001). SST data (grid cell of 16.5°N, 112.5°E) was downloaded from NOAA. <http://iridl.ldeo.columbia.edu/SOURCES/.NOAA/.NCEP/.EMC/.CMB/.GLOBAL/.Reyn_SmithOIv2/.monthly/.sst/>


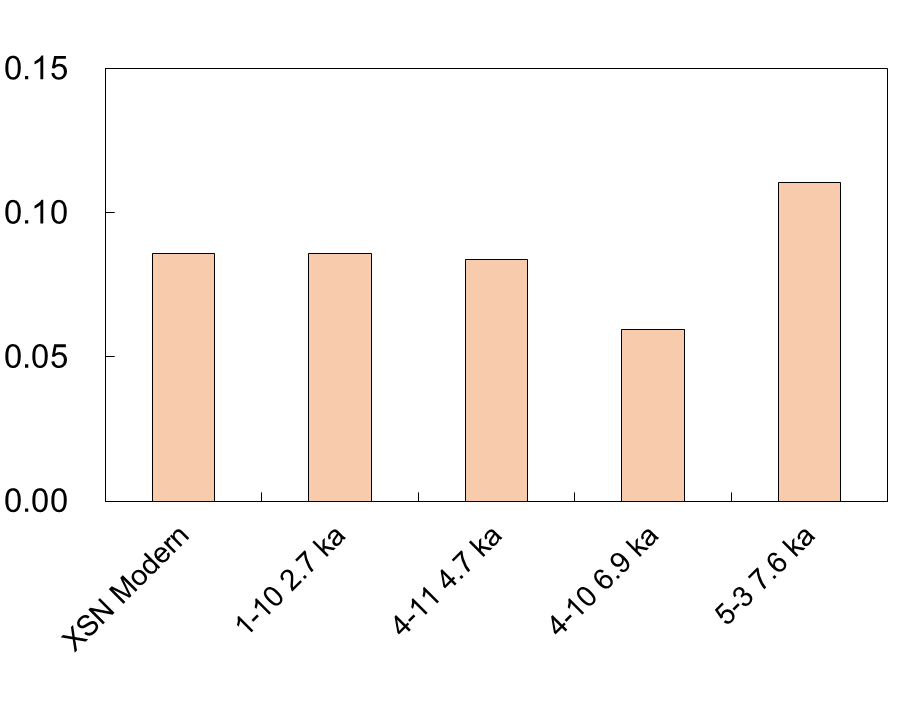


**Figure S4.** Standard deviation of bandpass filter results for various climatic records from this study.

**SI material 1**

The seasonal cycles seem not to be very clear for the modern and 7.6ka *Tridacna* sample, which is likely caused by the presence of several outliers of δ^18^O values. If we enlarge the figure of δ^18^O variation of the modern *Tridacana*, show the details of its variation pattern, and compare it with the instrumental SST record, the seasonal cycles are still very clear. The same situation also occurred for the 7.6ka *Tridacana* sample. We enlarged the figure of δ^18^O variation of the 7.6ka *Tridacana* sample to show the details of its variation pattern and the relative clear seasonal cycles.

**SI material 2**

As for the modern sample and sample 5-3 (7.6ka), the age models were mainly reconstructed based on yearly cyclicity visible by eyesight and comparison between the δ^18^O profile and the *Tridacna* annual banding. For parts of the modern sample, the seasonal cycle is clear. However, for other parts without obvious seasonal cycles, we compared it with the *Tridacna* annual banding (shown in the figure below), which could provide important information for reconstruction of age models. If the age models of any part still cannot be determined, we set two criteria to reconstruct the age models: 1. The maximum of the δ^18^O values within a small scope is assumed as the start of the year; 2. The number of the data points should be more than 6 values in each year cycle. Based on the above methods and criteria, the age models of the modern sample could be reconstructed. For the sample 5-3 (7.6ka), if we enlarge the figure of δ^18^O variation and show the details of its variation pattern (shown in the figure below), the seasonal cycles are still very clear.


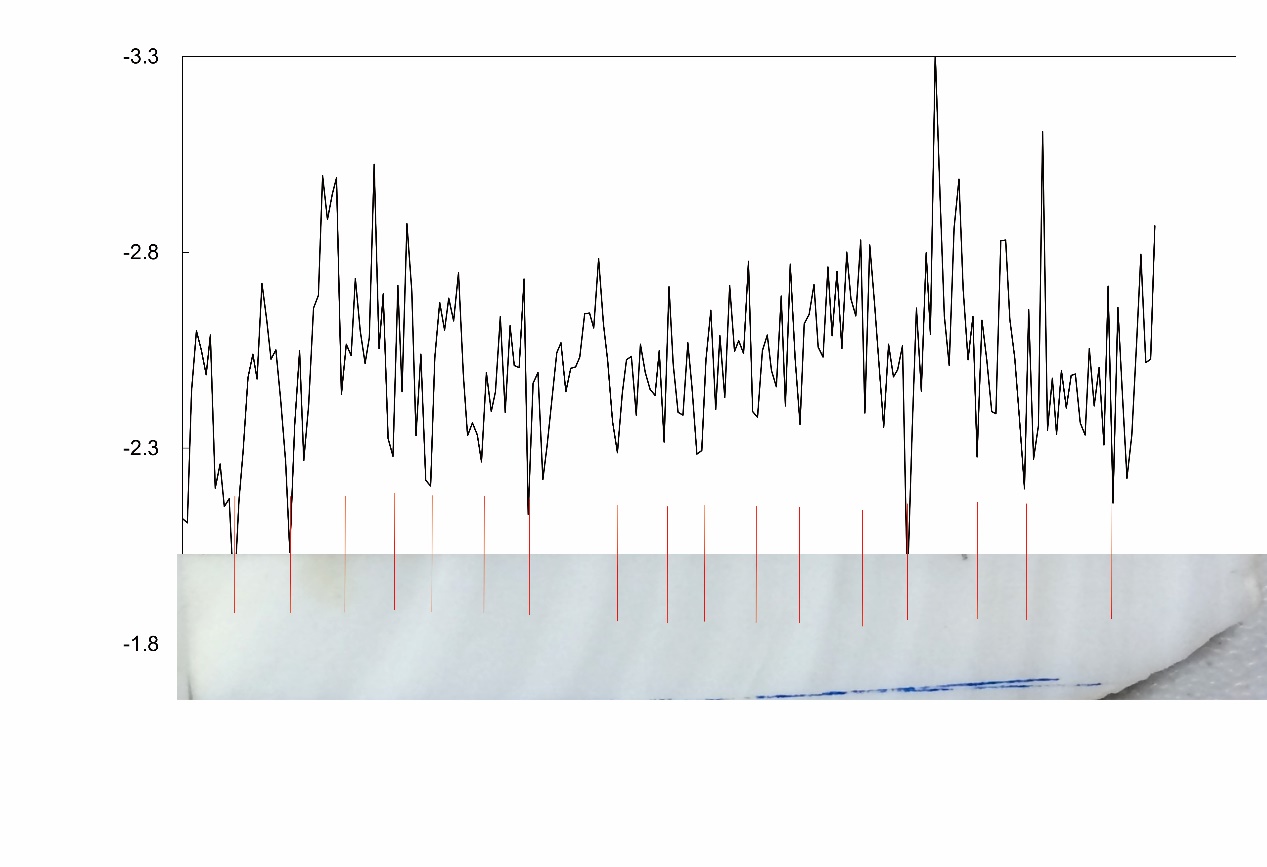


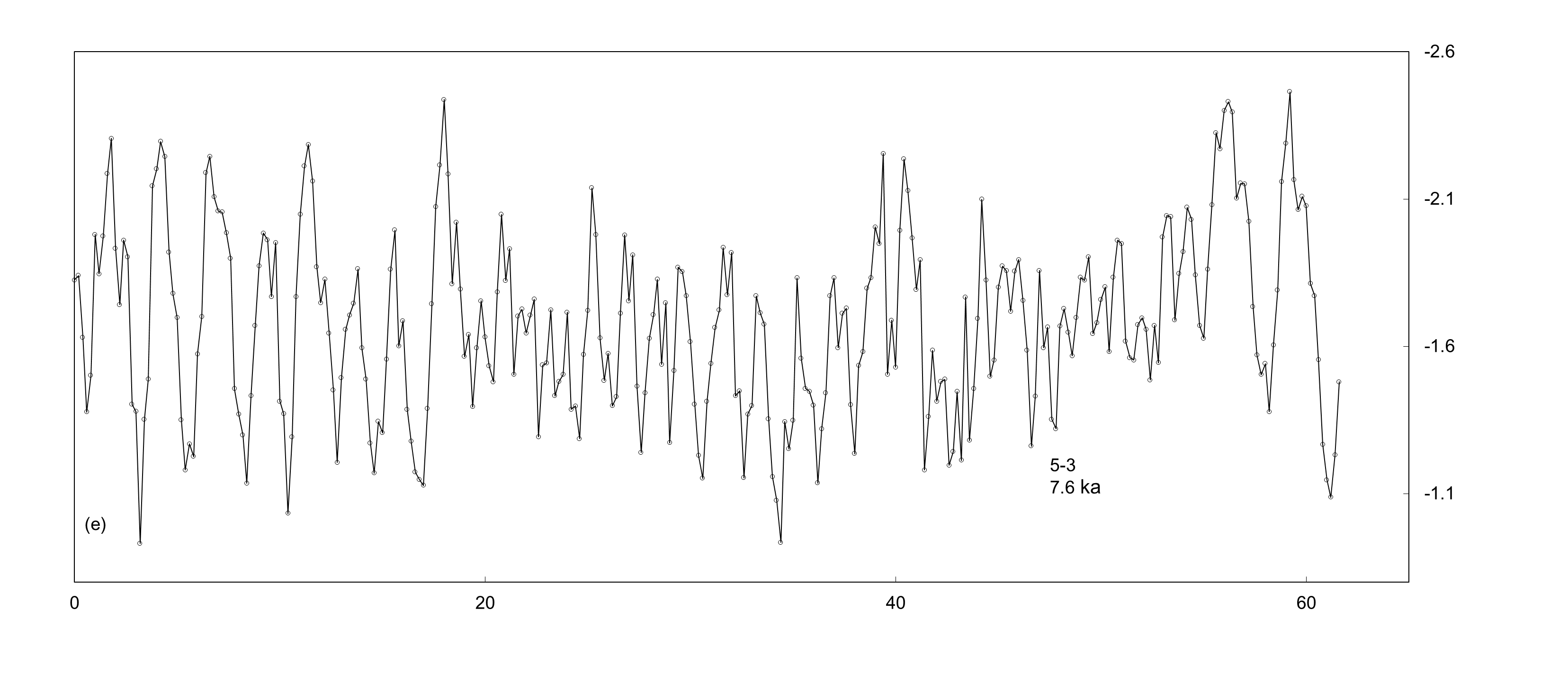

Supplement: Supplementary file 1 — Supplementary material. [file 41598_2020_61013_MOESM1_ESM.docx]
